# Comparison of negative pressure wound therapy with conventional wound care in the treatment of sternal wound infection after cardiac surgery: A meta-analysis with trial sequential analysis

**DOI:** 10.1371/journal.pone.0328771

**Published:** 2025-08-07

**Authors:** Si He, Na Tang, Sha Li

**Affiliations:** Hospital Infection Management Department, Changsha Stomatological Hospital, Stomatological Clinical College of Hunan University of Chinese Medicine, Changsha, China; Ataturk University Faculty of Medicine, TÜRKIYE

## Abstract

**Background:**

Negative pressure wound therapy (NPWT) has become a popular treatment option for sternal wound infection (SWI). However, it remains uncertain whether the therapeutic benefits of NPWT are superior to conventional wound care. This study aimed to systematically evaluate the therapeutic effects of NPWT on SWI compared to conventional wound care through meta-analysis.

**Methods:**

A comprehensive search of PubMed, Web of Science, Embase, and the Cochrane Library databases was conducted from inception to April 29, 2024 for all potential studies. The pooling of dichotomous outcome data was achieved using relative risk (RR), with results presented within a 95% confidence interval (CI). We utilized the standard mean difference (SMD) and 95% CI for continuous outcomes. Heterogeneity test, publication bias assessment, sensitivity analysis, and trial sequential analysis (TSA) were conducted. Publication bias was detected through the Begg’s and Egger’s tests. Software R 4.3.1, Stata 12.0, and TSA v0.9.5.10 Beta software were utilized for all analyses.

**Results:**

Out of 1832 articles identified, 10 were included in this study. The overall results revealed that NPWT significantly decreased the sternal wound reinfection (SWRI) rate (RR [95% CI] = 0.179 [0.099 to 0.323], 95% prediction interval [PI]: 0.082 to 0.442), in-hospital mortality (RR [95% CI] = 0.242 [0.149 to 0.394], 95% PI: 0.144 to 0.461), and shortened the length of intensive care unit (ICU) stay (SMD [95% CI] = −0.601 [−0.820 to −0.382], 95% PI: −1.317 to 0.128) compared with conventional wound care. There was no significant difference in length of hospital stay (SMD [95% CI] = −0.402 [−0.815 to 0.012], 95% PI: −1.801 to 0.998) and treatment duration (SMD [95% CI] = −0.398 [−1.646 to 0.849], 95% PI: −16.340 to 15.543) between the NPWT group and control group. Further subgroup analysis demonstrated the benefits of NPWT in shortening hospitalization length in the European population (*p* < 0.05).

**Conclusion:**

The present evidence corroborates that the application of NPWT in the treatment of SWI after cardiac surgery effectively reduces the SWRI incidence and in-hospital mortality while shortening the length of ICU stay.

## 1. Introduction

Cardiac surgery, often necessitating a sternotomy, serves as a life-saving intervention for numerous patients requiring procedures such as aortic root replacement, coronary artery bypass grafting, and valve repair or replacement. Concurrently, individuals who have undergone sternotomy are susceptible to sternal wound infection (SWI) [1]. Research suggested that the occurrence of SWI following cardiac surgery varies from 0.9% to 20% [2]. Critical instances of SWI can instigate organ failure impacting the heart, lung, and kidney, and can even result in death. The associated mortality rate ranges from 10% to 30% [3]. SWI is categorized into two types based on the severity and depth of the infection: superficial SWI (SSWI) which is limited to the subcutaneous tissue, skin, and deep fascia, and deep SWI (DSWI) which can impact sternum, muscle tissue, sub sternum, and mediastinum [4]. DSWI can delay recovery, prolong hospital stay, hinder functional status, and adversely affect a patient’s quality of life [5–7]. The most catastrophic outcomes of patients with SWI include osteomyelitis, sepsis, bypass graft erosion, ventricular rupture, and mediastinitis [8]. It is worth mentioning that the mortality associated with mediastinal infection remains significantly high, ranging from 3%−35% [9]. Therefore, immediate diagnosis and execution of efficient treatments for SWI are of utmost importance.

Conventional approaches to SWI management include re-closing the sternum after surgical debridement, wound debridement, and catheter irrigation of the wound bed using antimicrobial or antiseptic solutions [10]. Innovations in this field led to the adoption of open treatment and secondary closure. Traditionally, the wound bed has been loosely filled with gauze that not only soaks up the discharge and allows for air circulation but also maintains the necessary moisture to aid in the healing process. This gauze is routinely replaced every several days to promote the formation of a clean wound with healthy granulation tissue [11]. Conventional wet-to-dry dressings were employed to establish a sterile and damp environment that also helped to absorb surplus drainage from the wound. Yet, these dressings necessitated frequent replacements and could be uncomfortable to remove [12]. Addressing these challenges, Obdeijn et al. proposed the use of negative pressure wound therapy (NPWT) subsequent to SWI debridement [13]. NPWT, often referred to as vacuum-assisted closure, has markedly improved the management of open sternal wounds. The process of suctioning excess tissue fluid aids in preventing the formation of haematoma or seroma [14]. The application of negative pressure boosts perfusion, which in turn expedites the healing process. This approach also mitigates the risk of ischaemic wound necrosis, thereby preventing wound breakdown and promoting primary wound healing, particularly in watershed regions [15].

Several investigations have determined that NPWT is a reliable and beneficial approach to SWI in contrast to conventional methods [16,17]. Despite advancements in society guidelines regarding temperature management, preoperative shaving, glucose control, and prophylactic antibiotic administration to prevent infection prior to and during surgery, the evidence supporting the standard SWI management post-cardiac surgery remains a subject of debate. Furthermore, there is an absence of a comprehensive analysis comparing NPWT with conventional treatment for SWI. Consequently, we conducted a meta-analysis to systematically evaluate the effects of NPWT versus conventional wound care for SWI.

## 2. Methods

### 2.1 Study protocol

This study was executed in strict adherence to the Preferred Reporting Items for Systematic Reviews and Meta-Analyses (PRISMA) guidelines [18]. Prior to its commencement, the study’s protocol was duly registered with the International Prospective Register of Systematic Reviews (PROSPERO CRD42024537895).

### 2.2 Literature search

An exhaustive literature search was carried out on January 22, 2024, encompassing PubMed, Web of Science, Embase, and the Cochrane Library databases to identify all potential studies. The search was confined to English-language articles published from inception of each database to January 22, 2024. The following search items were used: (“negative pressure therapy” OR “negative pressure wound therapy” OR “negative-pressure wound therapy” OR “negative pressure wound treatment” OR “NPWT” OR “vacuum-assisted closure” OR “vacuum therapy”) AND (“sternotomy” OR “sternotomies” OR “sternal wound infection” OR “sternal wound-related infection” OR “mediastinitis” OR “mediastinum inflammation”). An updated literature search was conducted on April 29, 2024. To supplement this search, the reviewers scrutinized references within chosen articles and reviews. The detailed search strategy can be found in S1 File.

### 2.3 Study selection

Research was considered for inclusion if it satisfied the following conditions: (1) Cohort studies or randomized controlled trials (RCTs); (2) Studies exploring the therapeutic effects of NPWT on SWI; (3) Intervention arm: NPWT system, including vacuum assisted closure, vacuum sealing drainage, or topical negative pressure; (4) Control arm: conventional wound care, including conventional dressing therapy, closed or open irrigation, or open packing; (5) Outcomes: sternal wound reinfection (SWRI) rate, in-hospital mortality, length of hospital stay, intensive care unit (ICU) stay, and treatment duration. Reinfection was defined as the recurrence of infection after an initial infection-free period, evidenced by the resolution of clinical symptoms (e.g., absence of fever, normalized white blood cell count, and negative microbiological cultures). Criteria for exclusion included: (1) Case-control or single-arm studies; (2) The study population consisted of infants or children; (3) Studies without relevant outcomes; (4) Conference abstracts, reviews, letters and case reports.

### 2.4 Data extraction

Data and information from the included studies were independently extracted by two investigators. Any discordance between the two reviewers was rectified through deliberation or by involving a third reviewer. Excel spreadsheets served as the tool for data extraction. The information harvested from each study comprised the following elements: the lead author’s name along with the year of publication, research design, the country where the study was conducted, surgery type, sample size and age of participants, NPWT types, and outcomes. SWRI rate and in-hospital mortality were the primary outcomes; length of hospital stay, ICU stay, and treatment duration were the secondary outcomes.

### 2.5 Risk of bias assessment

The quality of the incorporated cohort research was assessed based on the Newcastle-Ottawa scale (NOS) [19]. Each study was rated on a scale of 0–9, with scores of 0–3, 4–6, and 7–9 indicating low, moderate, and high quality, respectively. We used the modified Jadad scale to assess the potential bias of RCTs [20]. Studies scoring between 0 and 3 were deemed low quality, whereas those with scores from 4 to 7 were considered high quality. The NOS and the modified Jadad scale were applied by two independent reviewers and any disputes were settled by consulting a third investigator.

### 2.6 Statistical analysis

The pooling of dichotomous outcome data was achieved using relative risk (RR) with results presented within a 95% confidence interval (CI). For continuous outcomes, we utilized the standard mean difference (SMD) and 95% CI. The Cochran’s Q statistic, Higgin’s I^2^ test, and 95% prediction interval (PI) were utilized to evaluate the overall heterogeneity of the study [21,22]. Heterogeneity was deemed acceptable if the *p* > 0.10 or I^2^ ≤ 50%. In the absence of significant heterogeneity, a fixed-effect model was chosen; otherwise, a random-effects model was applied [23]. To verify the robustness of the current analysis, we performed a sensitivity analysis using the leave-one-out method. Publication bias was determined through the Begg’s and Egger’s tests, supplemented by the visual interpretation of funnel plots [24,25]. The trim-and-fill method was chosen for quantitative adjustments in the presence of any publication bias [26]. All statistical analyses were conducted using Stata 12.0 and R software 4.3.1. A two-sided *p* < 0.05 was considered statistically significant.

### 2.7 Trial sequential analysis

In our study, a trial sequential analysis (TSA) was implemented to evaluate the robustness of the evidence and adjust for potential statistical errors [27]. Utilizing TSA software version 0.9.5.10 Beta (available at www.ctu.dk/tsa), we determined the required information size (RIS) along with the trial sequential monitoring boundaries. The establishment of O’Brien-Fleming α-spending boundaries involved a two-side approach with a preset 5% type I error rate and an 80% statistical power. The crossing of the cumulative Z-curve over either the RIS or the trial sequential monitoring boundary negated the need for additional research, thereby providing definitive evidence to either corroborate or refute the effect of the intervention.

## 3. Results

### 3.1 Study selection procedure

**Fig 1:**
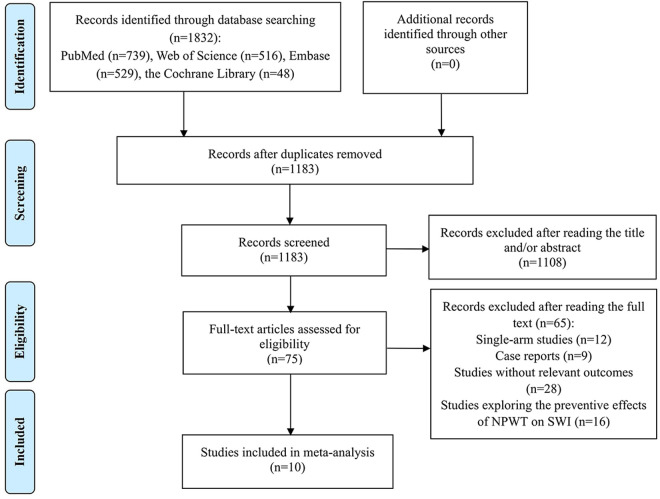
Flow diagram of the studies included in the meta-analysis.

Fig 1 outlines the selection process for the study. The initial search surfaced 1832 studies of potential relevance. Upon the removal of 649 repeated records, 1183 articles were left for review. These articles underwent a title/abstract screening, resulting in the dismissal of 1108 articles due to lack of relevance. A subsequent comprehensive review of the remaining 75 full texts led to the exclusion of 65 articles: 12 researches were single-arm studies; 9 articles were case reports; 28 studies failed to provide the required outcomes; and 16 studies explored the preventive effects of NPWT on SWI. Ultimately, 10 studies were included in the meta-analysis [11,12,28–35].

### 3.2 Study characteristics and quality assessment

Table 1 describes the characteristics of the studies incorporated into our analysis. Each of these studies, all of which were retrospective cohort studies, were conducted in various locations across Europe and Asia between the years 2010 and 2023. The group treated with NPWT comprised 469 participants, in contrast to the control group, which consisted of 413 individuals. 7 studies documented participants who had undergone sternotomy procedures, inclusive of median sternotomy. Conversely, 3 studies did not specify whether participants had undergone sternotomy, only indicating that these patients had been subjected to cardiac or open heart surgery. Additionally, the categories of SWI patients incorporated those with DSWI and mediastinitis. All studies have reported the therapeutic impacts of NPWT on SWI. The overall scores of these 10 retrospective cohort studies ranged from 7 to 9, indicating a low risk of bias. The detailed evaluation of study quality was provided in S1 Table in S2 File.

**Table 1:** Characteristics of the included studies.

First author (year)	Study design	Country	Surgery type	Sample size (E/C)	Age (E/C, mean ± SD, years)	NPWT group	Control group treatment	Types of SWI	Outcomes
Akbayrak (2023)	RCS	Turkey	Isolated coronary artery bypass graft operations via median sternotomy	52/62	68.4 ± 8.9/71.2 ± 9.3	VAC system, polyurethane foam, and special computer-controlled pump unit were used	Irrigation with povidone-iodine and saline solutions and open packing 3–4 times a day	Mediastinitis	2, 3, 5
De Feo (2011)	RCS	Italy	Median sternotomy	74/83	62.6 ± 10.8/59.2 ± 10.4	Sterile polyurethane foam dressing + VAC Therapy system	Washing pericardial sac with antibiotic and povidone-iodine solutions + Mediastinal irrigation alternating a 5% povidone-iodine solution with a 0.1% vancomycin solution every 6 hours	Mediastinitis	1, 2, 3, 4, 5
Vos (2012)	RCS	The Netherlands	Median sternotomy	89/24	67.9 ± 10.1/74.6 ± 8.4	VAC system, polyurethane foam, computer-controlled suction unit, tailored polyvinyl alcohol dressing were used	Wounds were irrigated with hydrogen peroxide, saline and diluted povidone-iodine solution, followed by packing with gauzes	Mediastinitis	2, 3, 4
Wang (2023)	RCS	China	Median sternotomy	16/18	63.8 ± 10.8/63.3 ± 13.0	Specialized system consisting of polyurethane foam and a computer‐controlled pump unit were utilized	Irrigation with povidone‐iodine and saline solutions, as well as open packing, were administered three to four times daily	Mediastinitis	3
Gegouskov (2022)	RCS	Bulgaria	Median sternotomy	32/45	66 ± 7/68 ± 8	Polyurethane sponge + Portable vacuum source	Thorough irrigation of the wound with saline or dilute povidone-iodine, debridement, and gauze dressing changes were performed	Deep SWI	1, 2
Risnes (2014)	RCS	Norway	Coronary artery bypass grafting through a median sternotomy	64/66	68.2 ± 9.2/63.3 ± 10.2	Sterile polyurethane sponge with an open-pore structure of 400–600 μm + Custom-built vacuum source	Open debridement, irrigation and Robicsek rewiring of the sternum	Mediastinitis	1
Steingrimsson (2012)	RCS	Iceland	Open heart surgery	20/23	69 ± 8/69 ± 10	Polyurethane foam + VAC system	Open dressings and/or closed irrigation	Deep SWI	1, 2, 3, 4
Petzina (2010)	RCS	Germany	Median sternotomy	69/49	NA	Polyurethane foam + Continuous vacuum source	Surgical debridement, removal of all sternal wires, drainage and irrigation, and re-stabilisation of the sternum	Mediastinitis	1, 2, 3
Saltarocchi (2023)	RCS	Italy	Cardiac surgery	19/15	63 (mean age)/69 (mean age)	White sterile polyurethane foam + Vacuum-assisted wound closure device	Closed catheter irrigation and subsequent sternal closure with the modified Robicsek technique	Deep SWI	2
Simek (2012)	RCS	Czech Republic	Open heart surgery	34/28	66.4 ± 9.8/71.2 ± 7.9	Topical negative pressure therapy with the strip of polyurethane foam and VAC system	Continuous irrigation with 2% povidone-iodine solution was started immediately throughout one indwelling substernal drain	Deep SWI	1, 2, 3, 4, 5

E, exposure group; C, control group; SD, standard deviation; NPWT, negative pressure wound therapy; RCS, retrospective cohort study; VAC, vacuum-assisted closure; SWI, sternal wound infection; NA, not available; 1, sternal wound reinfection rate; 2, in-hospital mortality; 3, hospital stay; 4, intensive care unit stay; 5, treatment duration.

### 3.3 Overall analysis of primary and secondary outcomes

**Fig 2:**
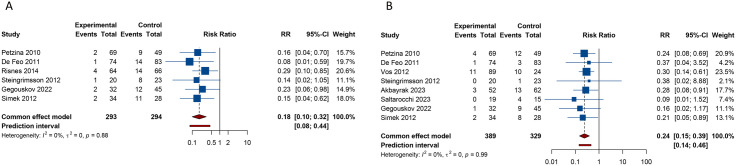
Forest plots of primary outcomes after negative pressure wound therapy versus conventional wound care for sternal wound infection. (A) Sternal wound reinfection rate; (B) In-hospital mortality.

There were 6 studies focused on the outcome of SWRI rate. Pooled results from the fixed-effects model (I^2^ = 0%, Tau^2^ = 0) indicated that NPWT significantly reduced the SWRI rate compared with the conventional wound care (RR [95% CI] = 0.179 [0.099 to 0.323], 95% PI: 0.082 to 0.442). A total of 8 studies furnished data on in-hospital mortality. The evidence, obtained from the fixed-effects model (I^2^ = 0%, Tau^2^ = 0), unveiled a lower in-hospital mortality when NPWT was employed, as opposed to conventional wound care (RR [95% CI] = 0.242 [0.149 to 0.394], 95% PI: 0.144 to 0.461) (Table 2, Fig 2).

**Table 2:** Pooled effect of the therapeutic effects of negative pressure wound therapy versus conventional wound care for sternal wound infection.

Outcomes	Number of studies	Meta-analysis				Heterogeneity	
		RR/SMD	95% CI	*p* value	95% PI	I^2^, Tau^2^	*p* value
SWRI rate	6	0.179	0.099, 0.323	<0.001	0.082, 0.442	0%, 0	0.883
In-hospital mortality	8	0.242	0.149, 0.394	<0.001	0.144, 0.461	0%, 0	0.989
ICU stay	4	−0.601	−0.820, −0.382	<0.001	−1.317, 0.128	18.1%, 0.0121	0.300
Hospital stay	7	−0.402	−0.815, 0.012	0.057	−1.801, 0.998	83.0%, 0.2519	<0.001
Treatment duration	3	−0.398	−1.646, 0.849	0.531	−16.340, 15.543	96.4%, 1.1691	<0.001

SWRI, sternal wound reinfection; RR, relative risk; SMD, standard mean difference; ICU, intensive care unit.

**Fig 3:**
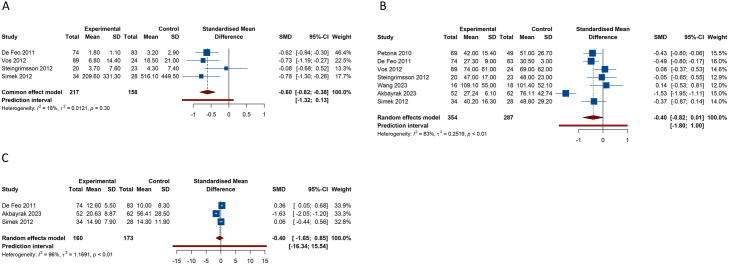
Forest plots of secondary outcomes after negative pressure wound therapy versus conventional wound care for sternal wound infection. (A) Intensive care unit stay; (B) Length of hospital stay; (C) Treatment duration.

4 studies addressed the outcome of ICU stay. No notable heterogeneity was discerned across these studies (I^2^ = 18.1%, Tau^2^ = 0.0121). Findings from the fixed-effects model indicated a significant decrease in ICU stay duration with NPWT in comparison to conventional wound care (SMD [95% CI] = −0.601 [−0.820 to −0.382], 95% PI: −1.317 to 0.128). There were 7 studies and 3 studies, focused on the outcomes of hospital stay length and treatment duration, respectively. Pooled results from the random-effects model (hospital stay: I^2^ = 83.0%, Tau^2^ = 0.2519; treatment duration: I^2^ = 96.4%, Tau^2^ = 1.1691) suggested that compared with conventional wound care, NPWT seem to shorten the length of hospital stay (SMD [95% CI] = −0.402 [−0.815 to 0.012], 95% PI: −1.801 to 0.998) and treatment duration (SMD [95% CI] = −0.398 [−1.646 to 0.849], 95% PI: −16.340 to 15.543), but without statistical significance (Table 2, Fig 3).

### 3.4 Subgroup analysis of primary and secondary outcomes

Subgroup analyses were performed only for groups with at least 2 included studies. Analysis stratified by patient ethnicity revealed that that NPWT significantly diminished SWRI rate (RR [95% CI] = 0.179 [0.099 to 0.323], 95% PI: 0.082 to 0.442; I^2^ = 0%, Tau^2^ = 0) and in-hospital mortality (RR [95% CI] = 0.235 [0.138 to 0.400], 95% PI: 0.131 to 0.494; I^2^ = 0%, Tau^2^ = 0), shortened the ICU stay (SMD [95% CI] = −0.601 [−0.820 to −0.382], 95% PI: −1.317 to 0.128; I^2^ = 18.1%, Tau^2^ = 0.0121) and length of hospital stay (SMD [95% CI] = −0.317 [−0.503 to −0.130], 95% PI: −0.825 to 0.226; I^2^ = 23.8%, Tau^2^ = 0.0148), and prolonged duration of treatment (SMD [95% CI] = 0.277 [0.010 to 0.544]; I^2^ = 1.1%, Tau^2^ = 0.0005) compared with conventional wound care in the European population (Table 3, S1–S5 Figs of S3 File).

**Table 3:** Subgroup analysis of the therapeutic effects of negative pressure wound therapy versus conventional wound care for sternal wound infection.

Outcomes and subgroups	Number of studies	Meta-analysis				Heterogeneity	
		RR/SMD	95% CI	*p* value	95% PI	I^2^, Tau^2^	*p* value
SWRI rate							
Subgrouped by ethnicity							
European	6	0.179	0.099, 0.323	<0.001	0.082, 0.442	0%, 0	0.883
Subgrouped by surgery types							
Median sternotomy	6	0.179	0.099, 0.323	<0.001	0.088, 0.415	0%, 0	0.883
Subgrouped by types of SWI							
Deep SWI	3	0.177	0.072, 0.433	<0.001	0.0005, 60.051	0%, 0	0.885
Mediastinitis	3	0.181	0.082, 0.397	<0.001	0.001-34.101	0%, 0	0.476
In-hospital mortality							
Subgrouped by ethnicity							
European	7	0.235	0.138, 0.400	<0.001	0.131, 0.494	0%, 0	0.972
Subgrouped by surgery types							
Median sternotomy	7	0.255	0.155, 0.418	<0.001	0.147, 0.478	0%, 0	0.995
Subgrouped by types of SWI							
Deep SWI	4	0.175	0.062, 0.489	0.001	0.019, 1.771	0%, 0	0.915
Mediastinitis	4	0.277	0.160, 0.478	<0.001	0.089, 0.885	0%, 0	0.980
ICU stay							
Subgrouped by ethnicity							
European	4	−0.601	−0.820, −0.382	<0.001	−1.317, 0.128	18.1%, 0.0121	0.300
Subgrouped by surgery types							
Median sternotomy	4	−0.601	−0.820, −0.382	<0.001	−1.129, −0.060	18.1%, 0.0121	0.300
Subgrouped by types of SWI							
Deep SWI	2	−0.445	−1.130, 0.240	0.203	–	66.5%, 0.1627	0.084
Mediastinitis	2	−0.656	−0.920, −0.393	<0.001	–	0%, 0	0.715
Hospital stay							
Subgrouped by ethnicity							
European	5	−0.317	−0.503, −0.130	0.001	−0.825, 0.226	23.8%, 0.0148	0.263
Subgrouped by surgery types							
Median sternotomy	7	−0.402	−0.815, 0.012	0.057	−1.734, 0.931	83.0%, 0.2519	<0.001
Subgrouped by types of SWI							
Deep SWI	2	−0.236	−0.622, 0.150	0.231	–	0%, 0	0.422
Mediastinitis	5	−0.466	−1.007, 0.076	0.092	−2.489, 1.557	87.8%, 0.3278	<0.001
Treatment duration							
Subgrouped by ethnicity							
European	2	0.277	0.010, 0.544	0.042	–	1.1%, 0.0005	0.315
Subgrouped by surgery types							
Median sternotomy	3	−0.398	−1.646, 0.849	0.531	−5.797, 5.000	96.4%, 1.1691	<0.001
Subgrouped by types of SWI							
Mediastinitis	2	−0.626	−2.574, 1.323	0.529	–	98.1%, 1.9406	<0.001

SWRI, sternal wound reinfection; RR, relative risk; SMD, standard mean difference; ICU, intensive care unit.

Categorizing the data by types of surgery, it was observed that for those patients who had a median sternotomy, NPWT notably lessened the SWRI rate (RR [95% CI] = 0.179 [0.099 to 0.323], 95% PI: 0.088 to 0.415; I^2^ = 0%, Tau^2^ = 0) and in-hospital mortality (RR [95% CI] = 0.255 [0.155 to 0.418], 95% PI: 0.147 to 0.478; I^2^ = 0%, Tau^2^ = 0), and abbreviated ICU stay (SMD [95% CI] = −0.601 [−0.820 to −0.382], 95% PI: −1.129 to −0.060; I^2^ = 0%, Tau^2^ = 0) compared to conventional wound care. However, no significant difference was observed in the length of hospital stay and treatment duration between the NPWT group and control group in patients who underwent median sternotomy (all *p* > 0.05) (Table 3, S1–S5 Figs of S3 File).

Upon classifying patients based on the specific type of SWI, the subgroup analysis results indicated that compared with conventional wound care, NPWT was found to significantly curtail the SWRI rate (RR [95% CI] = 0.177 [0.072 to 0.433], 95% PI: 0.0005 to 60.051; I^2^ = 0%, Tau^2^ = 0) and in-hospital mortality (RR [95% CI] = 0.175 [0.062 to 0.489], 95% PI: 0.019 to 1.771; I^2^ = 0%, Tau^2^ = 0) in patients with DSWI, but it did not notably alter the ICU stay and the length of hospital stay (all *p* > 0.05). Further subgroup analysis suggested that for the patients with mediastinitis, NPWT significantly diminished the SWRI rate (RR [95% CI] = 0.181 [0.082 to 0.397], 95% PI: 0.001 to 34.101; I^2^ = 0%, Tau^2^ = 0) and in-hospital mortality (RR [95% CI] = 0.277 [0.160 to 0.478], 95% PI: 0.089 to 0.885; I^2^ = 0%, Tau^2^ = 0), reduced the stay of ICU (SMD [95% CI] = −0.656 [−0.920 to −0.393]; I^2^ = 0%, Tau^2^ = 0), but had no effect on hospital stay and treatment duration (all *p* > 0.05) (Table 3, S1–S5 Figs of S3 File).

### 3.5 Trial sequential analysis results

**Fig 4:**
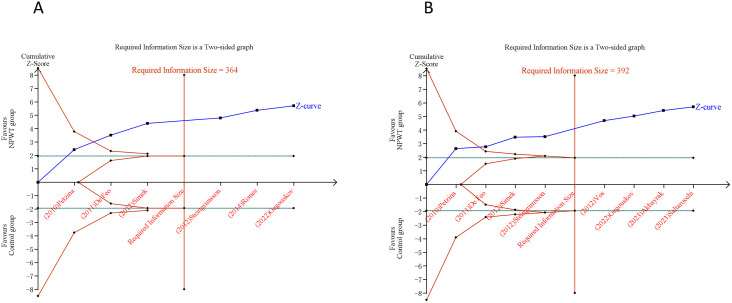
Trial sequential analysis of primary outcomes after negative pressure wound therapy versus conventional wound care for sternal wound infection. (A) Sternal wound reinfection rate; (B) In-hospital mortality. Uppermost and lowermost red curves represent trial sequential monitoring boundary lines for benefit and harm, respectively. Inner red lines represent the futility boundary. Blue line represents evolution of cumulative Z-score. Horizontal green lines represent the conventional boundaries for statistical significance. Cumulative Z-curve crossing the trial sequential monitoring boundary or the RIS boundary provides firm evidence of effect.

**Fig 5:**
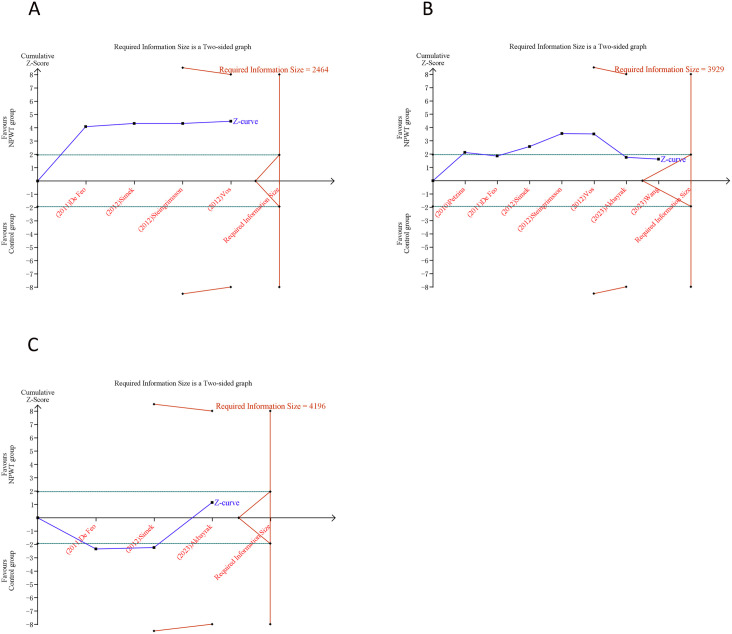
Trial sequential analysis of secondary outcomes after negative pressure wound therapy versus conventional wound care for sternal wound infection. (A) ICU stay; (B) Length of hospital stay; (C) Treatment duration. Uppermost and lowermost red curves represent trial sequential monitoring boundary lines for benefit and harm, respectively. Inner red lines represent the futility boundary. Blue line represents evolution of cumulative Z-score. Horizontal green lines represent the conventional boundaries for statistical significance. Cumulative Z-curve crossing the trial sequential monitoring boundary or the RIS boundary provides firm evidence of effect.

Fig 4 presented the trajectory of cumulative Z-curves pertaining to SWRI rate and in-hospital mortality, which intersect with both the RIS threshold and the trial sequential monitoring boundary. This convergence suggested a robust inference can be drawn regarding SWRI rate as well as in-hospital mortality. In contrast, the cumulative Z-curves corresponding to ICU stay, hospital stay and treatment duration failed to cross either the RIS boundary or the trial sequential monitoring boundary. This indicated that the capability to reach a definitive conclusion about ICU stay, hospital stay and treatment duration was somewhat constrained, potentially due to the existence of false positives (Fig 5).

### 3.6 Sensitivity analysis and publication bias

Sensitivity analysis and publication bias tests were exclusively executed for primary and secondary outcomes incorporating ≥ 6 studies. We employed a leave-one-out approach for sensitivity analysis to further verify the stability of our findings. The results suggested that Akbayrak et al.’s study may contribute to the high heterogeneity in the outcome of hospital stay length (S6 Fig of S3 File). Results from the Begg’s and Egger’s tests revealed that no significant publication bias existed in the results of in-hospital mortality and hospital stay. Nevertheless, potential publication bias might be present in the outcome related to SWRI rate (Begg’s test: *p* = 0.260, Egger’s test: *p* = 0.022). Further trim-and-fill technique was employed to adjust for publication bias. A comparison of the adjusted results with the original findings showed negligible differences, implying that the findings on SWRI rate maintain their reliability. The funnel plots were visualized in S7 Fig of S3 File.

## 4. Discussion

In the realm of SWI management, the application of NPWT has become increasingly prevalent. The technology behind NPWT involves specialized dressings connected to a device that generates negative pressure, uniformly applied across sealed post-surgical wounds [36,37]. A sterile, adhesive film seals the wound and adjacent skin, with a vacuum pump linked via a tube to create suction [37]. This system can administer pressures from −75 to −125 mmHg, facilitating the removal of wound fluids into a sterile container [38–40]. When contrasted with ordinary post-debridement drainage tube drainage, NPWT after wound infection has shown to enhance treatment outcomes, alleviate patient discomfort, and provide a sustained effect akin to ongoing debridement [4]. A systematic review has previously indicated that NPWT offers clinical advantages over other wound care strategies for SWI, including reduced hospitalization time, lower reinfection rates, and a decrease in early mortality [41]. Through a meta-analysis, our study demonstrated that compared with conventional wound care, NPWT notably decreased the incidence of SWRI and in-hospital mortality, as well as shortened the ICU stay in SWI patients. However, there were no significant differences in length of hospital stay and treatment duration between SWI patients receiving NPWT and those receiving conventional therapy.

NPWT can stimulate a hydrostatic pressure differential within the venous system, fostering improved blood circulation. This process aids in the efficient reduction of local osmotic active molecules, lessening tissue swelling, minimizing damage to the microcirculation, and preserving tissue blood supply. It also helps decrease residual irrigation fluid and inflammatory exudate, significantly alleviating patient discomfort. This mechanism potentially expedites patient recovery and minimizes the duration of hospital stays [28,39,42,43]. While our research has yet to confirm an advantage of NPWT over conventional wound care in reducing hospital length of stay, the pooled results suggested a trend toward shorter hospital stay, albeit without achieving statistical significance (SMD = −0.402, *p* = 0.057). Therefore, our findings regarding length of hospital stay may be subject to future refinement and updating as new studies are incorporated into the analysis. In addition, it has been proposed that the introduction of a sealed wound medium helps maintain the integrity of the sealed incision edges, spurring cellular proliferation and instigating angiogenesis [39,44,45]. This process drains extracellular fluid, facilitating the removal of exudative material and tissue edema. It also augments blood flow to the wound site, thereby enhancing tissue perfusion and boosting the circulation of antibiotics and immune cells [36,37,44]. These elements help thwart the advancement of infection and the emergence of sternal wound complications by inhibiting bacteria capable of forming colonies and promoting the generation of granulation tissue [39,44,45]. By inhibiting the advancement of infection and curtailing sternal wound complications, a reduction in the incidence of SWRI and in-hospital mortality among SWI patients can be achieved to a certain extent. This could explain the observed benefits of NPWT in reducing both SWRI rate and in-hospital mortality in our study. Furthermore, present studies showed that despite the relatively high expenses linked to NPWT equipment and care [46], its effectiveness in reducing hospital durations and enhancing clinical results affirms its cost-efficiency [47,48]. Further cost-effectiveness analyses also support NPWT as a therapeutic option offering substantial economic advantages [49].

Of note, our subgroup analysis results suggested that NPWT had a significant impact on reducing the SWRI rate, in-hospital mortality and ICU stay duration when compared to conventional treatment in patients with mediastinitis. Mediastinitis is identified as DSWIs accompanied by sternal osteomyelitis, with or without infection in the retrosternal space [50]. Diagnostic criteria for mediastinitis encompass sternal dehiscence, chest pain, purulent discharge, fever, and/or the isolation of microorganisms in mediastinal drainage cultures [51]. Sternal instability can contribute to the development of DSWI, which may be followed by skin deterioration and bacterial infiltration into deeper tissues. Initial treatment for mediastinitis involved surgical revisions accompanied by multiple open dressing changes, followed by either sternal rewiring or secondary healing. In cases where rewiring was not possible, muscle flaps were typically used. This treatment protocol was employed for an extended period, yet the mortality rate ranged from 10% to 47% as reported by various researchers [16,17,52]. The primary drawback of open dressings was thoracic instability, which was crucial for effective mechanical ventilation or spontaneous respiration. Prolonged immobilization increased the risk of further complications such as muscle weakness, thrombosis, and pneumonia [16]. Utilizing NPWT for mediastinitis situations has demonstrated advantageous results. These encompass an increase in parasternal blood circulation, a decrease in bacterial occupancy, and a hastened healing process of wounds owing to the development of granulation tissue [12]. Several researches have underscored the importance of NPWT in treating mediastinitis [34,52]. An extensive survey spanning 12 years, conducted by Lonie et al., disclosed a correlation between the use of NPWT and a reduced frequency of post-surgical complications requiring additional surgery post definitive wound sealing [53]. Remarkably, in their research, all patients treated with NPWT avoided the necessity for sternum rewiring, suggesting a supplementary benefit in stabilizing the sternum. Akbayrak et al.’s recent investigation demonstrated that the utilization of NPWT, in contrast to conventional treatment methods, led to notable reductions in metrics such as duration of hospital stay, overall treatment period, and in-hospital mortality rate [28]. The adoption of NPWT has lessened the requirement for intricate sternal closure procedures in patients with mediastinitis compared to conventional methods. This shift heralds the advent of more streamlined and economically efficient techniques [41,54].

Additionally, our subgroup analysis revealed a significant decrease in the occurrence of SWRI and in-hospital death rate in patients who had median sternotomy and were treated with NPWT, when compared to conventional wound care. Since several studies included in our analysis did not specify the type of sternotomy that SWI patients received, such as in the research conducted by Saltarocchi et al., where the subjects were identified as DSWI patients who had undergone cardiac surgery [32], it’s currently impossible to contrast the therapeutic impacts of NPWT on SWI across diverse sternotomy types. Therefore, additional updates for the subgroup analysis results based on different types of sternotomy in the future are warranted. Interestingly, NPWT was found to be associated with lower SWRI rate and in-hospital death rate, shorter ICU and hospital stays, and longer treatment duration in the European population. This observation is predominantly based on the fact that the majority of the studies included in our analysis were carried out in Europe. Only a single Asian study indicated no significant disparity between the NPWT group and conventional treatment group in terms of hospital stay duration [12]. Another investigation from Turkey revealed that the NPWT group experienced significant decreases in overall treatment duration, length of hospital stay, and in-hospital death rate compared to the group receiving conventional treatment [28]. These findings suggest potential regional variations in NPWT’s effectiveness, necessitating further research to elucidate the differential impact across diverse ethnic populations.

This study has some limitations. First, the challenges associated with implementing relevant RCTs allowed this study to combine findings only from retrospective cohort studies, which inevitably left our results subject to recall bias. Nevertheless, given the substantial advantages demonstrated by NPWT in existing evidence, conducting further RCTs may present ethical concerns. Specifically, randomly assigning patients to potentially less effective conventional treatments could expose them to unnecessary risks. Consequently, we recommend that further prospective cohort studies be conducted in specific contexts (e.g., resource-limited settings or the introduction of new technologies) to supplement the current evidence, rather than initiating new RCTs. Second, the majority of the included studies did not report the long-term outcomes of NPWT (e.g., quality of life and survival outcomes), hence, this study was confined to assessing the short-term effects of NPWT on SWI. The long-term implications of NPWT for SWI patients warrant further investigation. Additionally, more clinical indicators assessing the effect of NPWT on SWI, such as wound healing speed, deserve further exploration. Third, most of the studies included were conducted in Europe, with only one conducted in Asia, which somewhat limits the generalizability of our results to the global population. Fourth, TSA results suggested that additional studies and larger sample sizes are needed to obtain more stable and reliable results regarding ICU stay, length of hospital stay, and treatment duration.

## 5. Conclusion

In summary, our study indicated that compared with conventional wound care, NPWT notably decreased the incidence of SWRI, in-hospital mortality, and ICU stay. Further subgroup analyses suggested that NPWT could shorten the length of hospitalization in the European population.

## Supporting information

S1 Checklist PRISMA 2020 checklist. (PDF)

S1 File Search strategy. (DOCX)

S2 File Risk of bias and quality assessments for each study. (DOCX)

S3 File Subgroup analysis, sensitivity analysis and publication bias. (DOCX)

S1 Data Excluded studies with reasons for exclusion. (XLSX)

S2 Data Extracted data and used for analysis. (XLSX)

## References

[1] Fonseca MA, Cooper L. Reducing sternal wound infection rates in patients undergoing cardiothoracic surgery with sternotomy. Am J Nurs. 2024;124(4):48–54. doi: 10.1097/01.NAJ.0001010588.95227.5d 38511712

[2] Perezgrovas-Olaria R, Audisio K, Cancelli G, Rahouma M, Ibrahim M, Soletti GJ, et al. Deep sternal wound infection and mortality in cardiac surgery: a meta-analysis. Ann Thorac Surg. 2023;115(1):272–80. doi: 10.1016/j.athoracsur.2022.04.054 35618048

[3] Kaye AE, Kaye AJ, Pahk B, McKenna ML, Low DW. Sternal wound reconstruction: management in different cardiac populations. Ann Plast Surg. 2010;64(5):658–66. doi: 10.1097/SAP.0b013e3181dba841 20395796

[4] Song Y, Chu W, Sun J, Liu X, Zhu H, Yu H, et al. Review on risk factors, classification, and treatment of sternal wound infection. J Cardiothorac Surg. 2023;18(1):184. doi: 10.1186/s13019-023-02228-y 37208736 PMC10199640

[5] Arribas-Leal JM, Rivera-Caravaca JM, Hernández-Torres A, Jiménez-Aceituna A, Moral-Escudero E, Pérez-Andreu J, et al. Incidence and predictors of sternal surgical wound infection in cardiac surgery: a prospective study. Int Wound J. 2023;20(4):917–24. doi: 10.1111/iwj.13938 36168924 PMC10031248

[6] Locke T, Parsons H, Briffa N, Stott M, de Silva TI, Darton TC. A bundle of infection control measures reduces postoperative sternal wound infection due to Staphylococcus aureus but not Gram-negative bacteria: a retrospective analysis of 6903 patient episodes. J Hosp Infect. 2022;126:21–8. doi: 10.1016/j.jhin.2022.03.006 35341810

[7] Phoon PHY, Hwang NC. Deep sternal wound infection: diagnosis, treatment and prevention. J Cardiothorac Vasc Anesth. 2020;34(6):1602–13. doi: 10.1053/j.jvca.2019.09.019 31623967

[8] Hamaguchi R, Shekar PS, Johnson JA, Orgill DP. Current management of sternal wounds. Plast Reconstr Surg. 2021;148(6):1012e–25e. doi: 10.1097/PRS.0000000000008510 34847131

[9] Mekontso Dessap A, Vivier E, Girou E, Brun-Buisson C, Kirsch M. Effect of time to onset on clinical features and prognosis of post-sternotomy mediastinitis. Clin Microbiol Infect. 2011;17(2):292–9. doi: 10.1111/j.1469-0691.2010.03197.x 20167008

[10] Ambe PC, Rombey T, Rembe J-D, Dörner J, Zirngibl H, Pieper D. The role of saline irrigation prior to wound closure in the reduction of surgical site infection: a systematic review and meta-analysis. Patient Saf Surg. 2020;14(1):47. doi: 10.1186/s13037-020-00274-2 33353558 PMC7756962

[11] Gegouskov V, Manchev G, Goranovska V, Stoykov D. Negative pressure wound therapy becomes the treatment of choice of deep sternal wound infection. Heart Surg Forum. 2022;25(4):E601–7. doi: 10.1532/hsf.4791 36052909

[12] Wang W-T, Lee J-M, Chiang K-J, Chiou S-H, Wang C-T, Wu S-H. The role of negative pressure wound therapy in the treatment of poststernotomy mediastinitis in Asians: a single-center, retrospective cohort study. Health Sci Rep. 2023;6(11):e1675. doi: 10.1002/hsr2.1675 38028682 PMC10644291

[13] Obdeijn MC, de Lange MY, Lichtendahl DH, de Boer WJ. Vacuum-assisted closure in the treatment of poststernotomy mediastinitis. Ann Thorac Surg. 1999;68(6):2358–60. doi: 10.1016/s0003-4975(99)01159-5 10617044

[14] Dohmen PM, Misfeld M, Borger MA, Mohr FW. Closed incision management with negative pressure wound therapy. Expert Rev Med Devices. 2014;11(4):395–402. doi: 10.1586/17434440.2014.911081 24754343

[15] Atkins BZ, Tetterton JK, Petersen RP, Hurley K, Wolfe WG. Laser Doppler flowmetry assessment of peristernal perfusion after cardiac surgery: beneficial effect of negative pressure therapy. Int Wound J. 2011;8(1):56–62. doi: 10.1111/j.1742-481X.2010.00743.x 21167000 PMC7950932

[16] Sjögren J, Gustafsson R, Nilsson J, Malmsjö M, Ingemansson R. Clinical outcome after poststernotomy mediastinitis: vacuum-assisted closure versus conventional treatment. Ann Thorac Surg. 2005;79(6):2049–55. doi: 10.1016/j.athoracsur.2004.12.048 15919308

[17] Fuchs U, Zittermann A, Stuettgen B, Groening A, Minami K, Koerfer R. Clinical outcome of patients with deep sternal wound infection managed by vacuum-assisted closure compared to conventional therapy with open packing: a retrospective analysis. Ann Thorac Surg. 2005;79(2):526–31. doi: 10.1016/j.athoracsur.2004.08.032 15680828

[18] Page MJ, McKenzie JE, Bossuyt PM, Boutron I, Hoffmann TC, Mulrow CD, et al. The PRISMA 2020 statement: an updated guideline for reporting systematic reviews. BMJ. 2021;372:n71. doi: 10.1136/bmj.n71 33782057 PMC8005924

[19] Wells GA, Shea B, O’Connell J. The Newcastle-Ottawa Scale (NOS) for assessing the quality of nonrandomised studies in meta-analyses. 2014.

[20] Jadad AR, Moore RA, Carroll D, Jenkinson C, Reynolds DJ, Gavaghan DJ, et al. Assessing the quality of reports of randomized clinical trials: is blinding necessary? Control Clin Trials. 1996;17(1):1–12. doi: 10.1016/0197-2456(95)00134-4 8721797

[21] Bowden J, Tierney JF, Copas AJ, Burdett S. Quantifying, displaying and accounting for heterogeneity in the meta-analysis of RCTs using standard and generalised Q statistics. BMC Med Res Methodol. 2011;11:41. doi: 10.1186/1471-2288-11-41 21473747 PMC3102034

[22] IntHout J, Ioannidis JPA, Rovers MM, Goeman JJ. Plea for routinely presenting prediction intervals in meta-analysis. BMJ Open. 2016;6(7):e010247. doi: 10.1136/bmjopen-2015-010247 27406637 PMC4947751

[23] Higgins JPT, Thompson SG. Quantifying heterogeneity in a meta-analysis. Stat Med. 2002;21(11):1539–58. doi: 10.1002/sim.1186 12111919

[24] Egger M, Davey Smith G, Schneider M, Minder C. Bias in meta-analysis detected by a simple, graphical test. BMJ. 1997;315(7109):629–34. doi: 10.1136/bmj.315.7109.629 9310563 PMC2127453

[25] Begg CB, Mazumdar M. Operating characteristics of a rank correlation test for publication bias. Biometrics. 1994;50(4):1088–101. doi: 10.2307/2533446 7786990

[26] Duval S, Tweedie R. Trim and fill: A simple funnel-plot-based method of testing and adjusting for publication bias in meta-analysis. Biometrics. 2000;56(2):455–63. doi: 10.1111/j.0006-341x.2000.00455.x 10877304

[27] Wetterslev J, Jakobsen JC, Gluud C. Trial sequential analysis in systematic reviews with meta-analysis. BMC Med Res Methodol. 2017;17(1):39. doi: 10.1186/s12874-017-0315-7 28264661 PMC5397700

[28] Akbayrak H, Tekumit H. Comparison between vacuum-assisted closure technique and conventional approach in patients with mediastinitis after isolated coronary artery bypass graft surgery. Braz J Cardiovasc Surg. 2023;38(3):353–9. doi: 10.21470/1678-9741-2022-0317 36692043 PMC10159076

[29] De Feo M, Della Corte A, Vicchio M, Pirozzi F, Nappi G, Cotrufo M. Is post-sternotomy mediastinitis still devastating after the advent of negative-pressure wound therapy? Tex Heart Inst J. 2011;38(4):375–80. 21841864 PMC3147206

[30] Petzina R, Hoffmann J, Navasardyan A, Malmsjö M, Stamm C, Unbehaun A, et al. Negative pressure wound therapy for post-sternotomy mediastinitis reduces mortality rate and sternal re-infection rate compared to conventional treatment. Eur J Cardiothorac Surg. 2010;38(1):110–3. doi: 10.1016/j.ejcts.2010.01.028 20171898

[31] Risnes I, Abdelnoor M, Veel T, Svennevig JL, Lundblad R, Rynning SE. Mediastinitis after coronary artery bypass grafting: the effect of vacuum-assisted closure versus traditional closed drainage on survival and re-infection rate. Int Wound J. 2014;11(2):177–82. doi: 10.1111/j.1742-481X.2012.01060.x 22925188 PMC7950556

[32] Saltarocchi S, Chourda E, D’Abramo M, Saade W, Miraldi F. Vacuum-assisted wound closure with instillation followed by nitinol clips application to treat deep sternal wound infections after cardiac surgery: evolution of a two-step approach. Wounds. 2023;35(2):E63–8. doi: 10.25270/wnds/21141 36897616

[33] Simek M, Hajek R, Fluger I, Molitor M, Grulichova J, Langova K, et al. Superiority of topical negative pressure over closed irrigation therapy of deep sternal wound infection in cardiac surgery. J Cardiovasc Surg (Torino). 2012;53(1):113–20. 22231537

[34] Steingrimsson S, Gottfredsson M, Gudmundsdottir I, Sjögren J, Gudbjartsson T. Negative-pressure wound therapy for deep sternal wound infections reduces the rate of surgical interventions for early re-infections. Interact Cardiovasc Thorac Surg. 2012;15(3):406–10. doi: 10.1093/icvts/ivs254 22691377 PMC3422957

[35] Vos RJ, Yilmaz A, Sonker U, Kelder JC, Kloppenburg GTL. Vacuum-assisted closure of post-sternotomy mediastinitis as compared to open packing. Interact Cardiovasc Thorac Surg. 2012;14(1):17–21. doi: 10.1093/icvts/ivr049 22108946 PMC3420287

[36] Dohmen PM, Markou T, Ingemansson R, Rotering H, Hartman JM, van Valen R, et al. Use of incisional negative pressure wound therapy on closed median sternal incisions after cardiothoracic surgery: clinical evidence and consensus recommendations. Med Sci Monit. 2014;20:1814–25. doi: 10.12659/MSM.891169 25280449 PMC4199398

[37] Scalise A, Calamita R, Tartaglione C, Pierangeli M, Bolletta E, Gioacchini M, et al. Improving wound healing and preventing surgical site complications of closed surgical incisions: a possible role of Incisional Negative Pressure Wound Therapy. A systematic review of the literature. Int Wound J. 2016;13(6):1260–81. doi: 10.1111/iwj.12492 26424609 PMC7950088

[38] Atkins BZ, Wooten MK, Kistler J, Hurley K, Hughes GC, Wolfe WG. Does negative pressure wound therapy have a role in preventing poststernotomy wound complications? Surg Innov. 2009;16(2):140–6. doi: 10.1177/1553350609334821 19460818

[39] Gatti G, Ledwon M, Gazdag L, Cuomo F, Pappalardo A, Fischlein T, et al. Management of closed sternal incision after bilateral internal thoracic artery grafting with a single-use negative pressure system. Updates Surg. 2018;70(4):545–52. doi: 10.1007/s13304-018-0515-7 29460174

[40] Colli A, Camara M-L. First experience with a new negative pressure incision management system on surgical incisions after cardiac surgery in high risk patients. J Cardiothorac Surg. 2011;6:160. doi: 10.1186/1749-8090-6-160 22145641 PMC3305521

[41] Yu AW, Rippel RA, Smock E, Jarral OA. In patients with post-sternotomy mediastinitis is vacuum-assisted closure superior to conventional therapy? Interact Cardiovasc Thorac Surg. 2013;17(5):861–5. doi: 10.1093/icvts/ivt326 23912622 PMC3805210

[42] Drossos G, Ampatzidou F, Baddour A, Madesis A, Karaiskos T. The impact of deep sternal wound infections treated by negative pressure on early, 1 year and late mortality: a longitudinal case-control study. J Card Surg. 2019;34(12):1550–5. doi: 10.1111/jocs.14296 31654592

[43] Gatti G, Benussi B, Brunetti D, Ceschia A, Porcari A, Biondi F, et al. The fate of patients having deep sternal infection after bilateral internal thoracic artery grafting in the negative pressure wound therapy era. Int J Cardiol. 2018;269:67–74. doi: 10.1016/j.ijcard.2018.07.090 30049494

[44] Grauhan O, Navasardyan A, Hofmann M, Müller P, Stein J, Hetzer R. Prevention of poststernotomy wound infections in obese patients by negative pressure wound therapy. J Thorac Cardiovasc Surg. 2013;145(5):1387–92. doi: 10.1016/j.jtcvs.2012.09.040 23111014

[45] Strugala V, Martin R. Meta-analysis of comparative trials evaluating a prophylactic single-use negative pressure wound therapy system for the prevention of surgical site complications. Surg Infect (Larchmt). 2017;18(7):810–9. doi: 10.1089/sur.2017.156 28885895 PMC5649123

[46] Souza SC de, Mendes CMC, Meneses JVL, Dias RM. Simplified vacuum dressing system: effectiveness and safety in wounds management. Acta Cir Bras. 2022;37(9):e370906. doi: 10.1590/acb370906 36515315 PMC9746547

[47] Muresan M, Suciu H, Marian D, Muresan AV, Gabor RM, Neagoe RM, et al. A single-center prospective study on the efficiency of negative pressure wound therapy versus conventional wound therapy in the postoperative management of devitalized and infected wounds. Ann Ital Chir. 2023;94:411–8. 37794810

[48] Nherera LM, Saunders C, Verma S, Trueman P, Fatoye F. Single-use negative pressure wound therapy reduces costs in closed surgical incisions: UK and US economic evaluation. J Wound Care. 2021;30(Sup5):S23–31. doi: 10.12968/jowc.2021.30.Sup5.S23 33979232

[49] Hawkins RB, Mehaffey JH, Charles EJ, Krebs ED, Smith JG, Kern JA, et al. Cost-effectiveness of negative pressure incision management system in cardiac surgery. J Surg Res. 2019;240:227–35. doi: 10.1016/j.jss.2019.02.046 30999239 PMC6536336

[50] Bouza E, de Alarcón A, Fariñas MC, Gálvez J, Goenaga MÁ, Gutiérrez-Díez F, et al. Prevention, Diagnosis and Management of Post-Surgical Mediastinitis in Adults Consensus Guidelines of the Spanish Society of Cardiovascular Infections (SEICAV), the Spanish Society of Thoracic and Cardiovascular Surgery (SECTCV) and the Biomedical Research Centre Network for Respiratory Diseases (CIBERES). J Clin Med. 2021;10(23):5566. doi: 10.3390/jcm10235566 34884268 PMC8658224

[51] Garner JS, Jarvis WR, Emori TG, Horan TC, Hughes JM. CDC definitions for nosocomial infections, 1988. Am J Infect Control. 1988;16(3):128–40. doi: 10.1016/0196-6553(88)90053-3 2841893

[52] Raja SG, Berg GA. Should vacuum-assisted closure therapy be routinely used for management of deep sternal wound infection after cardiac surgery? Interact Cardiovasc Thorac Surg. 2007;6(4):523–7. doi: 10.1510/icvts.2007.157370 17669926

[53] Lonie S, Hallam J, Yii M, Davis P, Newcomb A, Nixon I, et al. Changes in the management of deep sternal wound infections: a 12-year review. ANZ J Surg. 2015;85(11):878–81. doi: 10.1111/ans.13279 26331481

[54] Vos RJ, Yilmaz A, Sonker U, Kelder JC, Kloppenburg GTL. Primary closure using Redon drains vs vacuum-assisted closure in post-sternotomy mediastinitis. Eur J Cardiothorac Surg. 2012;42(4):e53-7. doi: 10.1093/ejcts/ezs404 22885227

