# Supplementary material for: Comparison of negative pressure wound therapy with conventional wound care in the treatment of sternal wound infection after cardiac surgery: A meta-analysis with trial sequential analysis
Source: PLoS One. 2025 Aug 7;20(8):e0328771. doi: 10.1371/journal.pone.0328771 (PMC12331072; doi:10.1371/journal.pone.0328771)
Supplement: S1 File — (DOCX) [file pone.0328771.s002.docx]

**PubMed 739**

#1 (negative pressure therapy) OR (negative pressure wound therapy) OR (negative-pressure wound therapy) OR (negative pressure wound treatment) OR NPWT OR (vacuum-assisted closure) OR (vacuum assisted closure) OR (vacuum therapy)

#2 (sternotomy OR sternotomies) OR (sternal wound infection) OR (sternal wound-related infection) OR (mediastinitis OR mediastinum inflammation)

#3 #1 AND #2

**Web of Science 516**

#1 TS=((negative pressure therapy) OR (negative pressure wound therapy) OR (negative-pressure wound therapy) OR (negative pressure wound treatment) OR NPWT OR (vacuum-assisted closure) OR (vacuum assisted closure) OR (vacuum therapy))

#2 TS=((sternotomy OR sternotomies) OR (sternal wound infection) OR (sternal wound-related infection) OR (mediastinitis OR mediastinum inflammation))

#3 #1 AND #2

**Embase 529**

#1 'negative pressure therapy'/exp OR 'negative pressure wound therapy'/exp OR 'negative-pressure wound therapy'/exp OR 'negative pressure wound treatment' OR npwt OR 'vacuum-assisted closure'/exp OR 'vacuum assisted closure'/exp OR 'vacuum therapy'/exp

#2 'sternotomy'/exp OR sternotomies OR 'sternal wound infection'/exp OR 'sternal wound-related infection' OR 'mediastinitis'/exp OR 'mediastinum inflammation'

#3 #1 AND #2

**The Cochrane Library 48**

#1 All Text=((negative pressure therapy) OR (negative pressure wound therapy) OR (negative-pressure wound therapy) OR (negative pressure wound treatment) OR NPWT OR (vacuum-assisted closure) OR (vacuum assisted closure) OR (vacuum therapy))

#2 All Text=((sternotomy OR sternotomies) OR (sternal wound infection) OR (sternal wound-related infection) OR (mediastinitis OR mediastinum inflammation))

#3 #1 AND #2
