# Supplementary material for: Comparison of negative pressure wound therapy with conventional wound care in the treatment of sternal wound infection after cardiac surgery: A meta-analysis with trial sequential analysis
Source: PLoS One. 2025 Aug 7;20(8):e0328771. doi: 10.1371/journal.pone.0328771 (PMC12331072; doi:10.1371/journal.pone.0328771)
Supplement: S2 File — (DOCX) [file pone.0328771.s003.docx]

| **Table S1.** The Newcastle-Ottawa quality assessment scale of the included cohort studies. | | | | | | | | | | | | |
| --- | --- | --- | --- | --- | --- | --- | --- | --- | --- | --- | --- | --- |
| Study | Selection | | | |  | Comparability | |  | Assessment of outcome | | | Total score |
|  | Representativeness of exposure arm(s) | Selection of the comparative arm(s) | Origin of exposure source | Demonstration that outcome of interest was not present at start of study |  | Studies controlling the most important factors | Studies controlling the other main factors |  | Assessment of outcome with independency | Adequacy of follow-up length | Lost to follow-up acceptable |  |
| Petzina (2010) | 1 | 1 | 1 | 1 |  | 1 | 1 |  | 1 | 0 | 1 | 8 |
| De Feo (2011) | 1 | 1 | 1 | 1 |  | 1 | 1 |  | 1 | 0 | 1 | 8 |
| Vos (2012) | 1 | 1 | 1 | 1 |  | 1 | 0 |  | 1 | 0 | 1 | 7 |
| Risnes (2014) | 1 | 1 | 1 | 1 |  | 1 | 0 |  | 1 | 1 | 1 | 8 |
| Steingrimsson (2012) | 1 | 1 | 1 | 1 |  | 1 | 1 |  | 1 | 1 | 1 | 9 |
| Wang (2023) | 1 | 1 | 1 | 1 |  | 1 | 1 |  | 1 | 0 | 1 | 8 |
| Akbayrak (2023) | 1 | 1 | 1 | 1 |  | 1 | 1 |  | 1 | 0 | 1 | 8 |
| Saltarocchi (2023) | 1 | 1 | 1 | 1 |  | 1 | 0 |  | 1 | 0 | 1 | 7 |
| Gegouskov (2022) | 1 | 1 | 1 | 1 |  | 1 | 1 |  | 1 | 0 | 1 | 8 |
| Simek (2012) | 1 | 1 | 1 | 1 |  | 1 | 1 |  | 1 | 0 | 1 | 8 |
