# Supplementary material for: Comparison of negative pressure wound therapy with conventional wound care in the treatment of sternal wound infection after cardiac surgery: A meta-analysis with trial sequential analysis
Source: PLoS One. 2025 Aug 7;20(8):e0328771. doi: 10.1371/journal.pone.0328771 (PMC12331072; doi:10.1371/journal.pone.0328771)

**Fig S1.** Subgroup analysis of sternal wound reinfection rate after negative pressure wound therapy versus conventional wound care for sternal wound infection (SWI). (A) Subgroup = European; (B) Subgroup = Median sternotomy; (C) Subgroup = Deep SWI; (D) Subgroup = Mediastinitis.


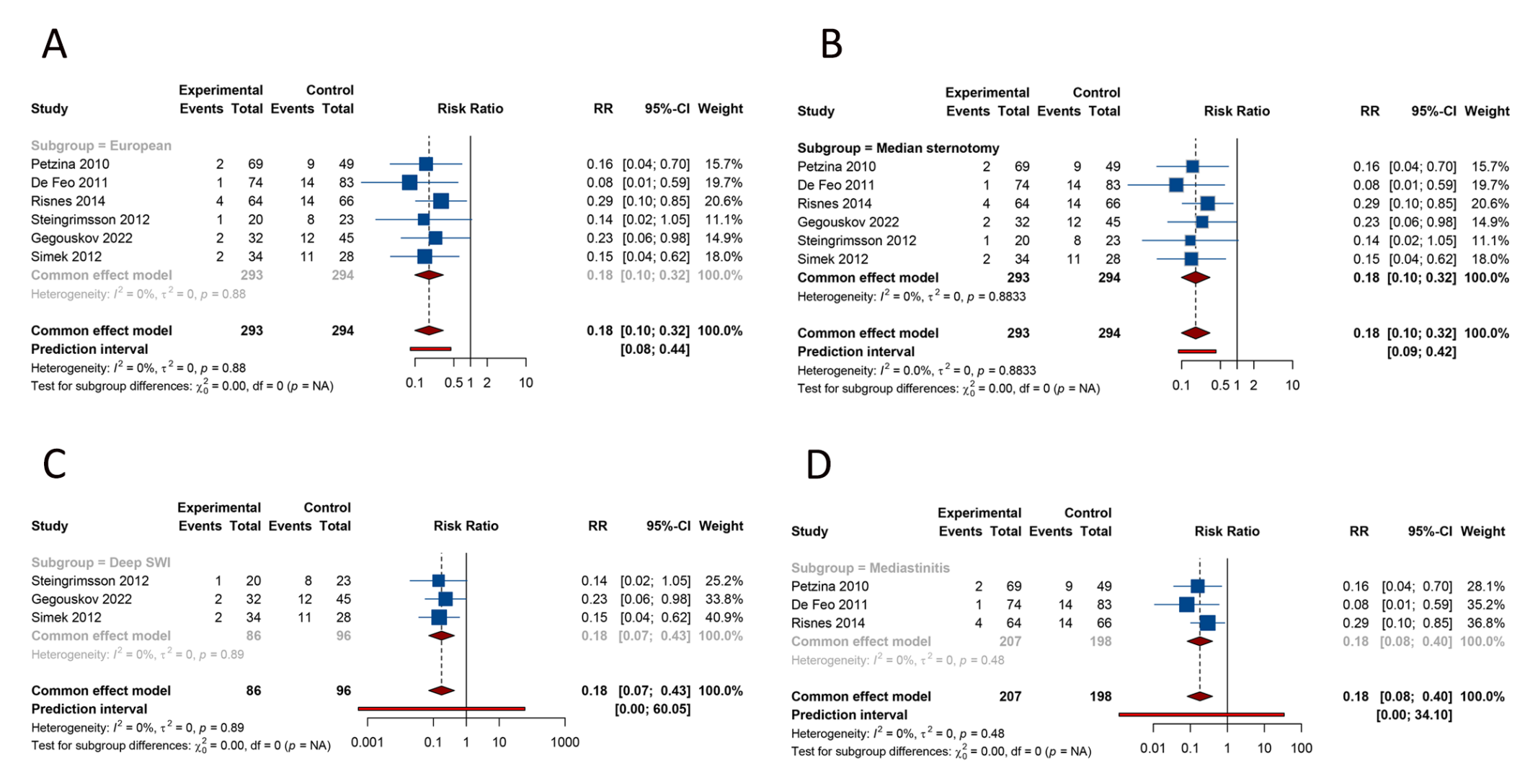


**Fig S2.** Subgroup analysis of in-hospital mortality after negative pressure wound therapy versus conventional wound care for sternal wound infection (SWI). (A) Subgroup = European; (B) Subgroup = Median sternotomy; (C) Subgroup = Deep SWI; (D) Subgroup = Mediastinitis.


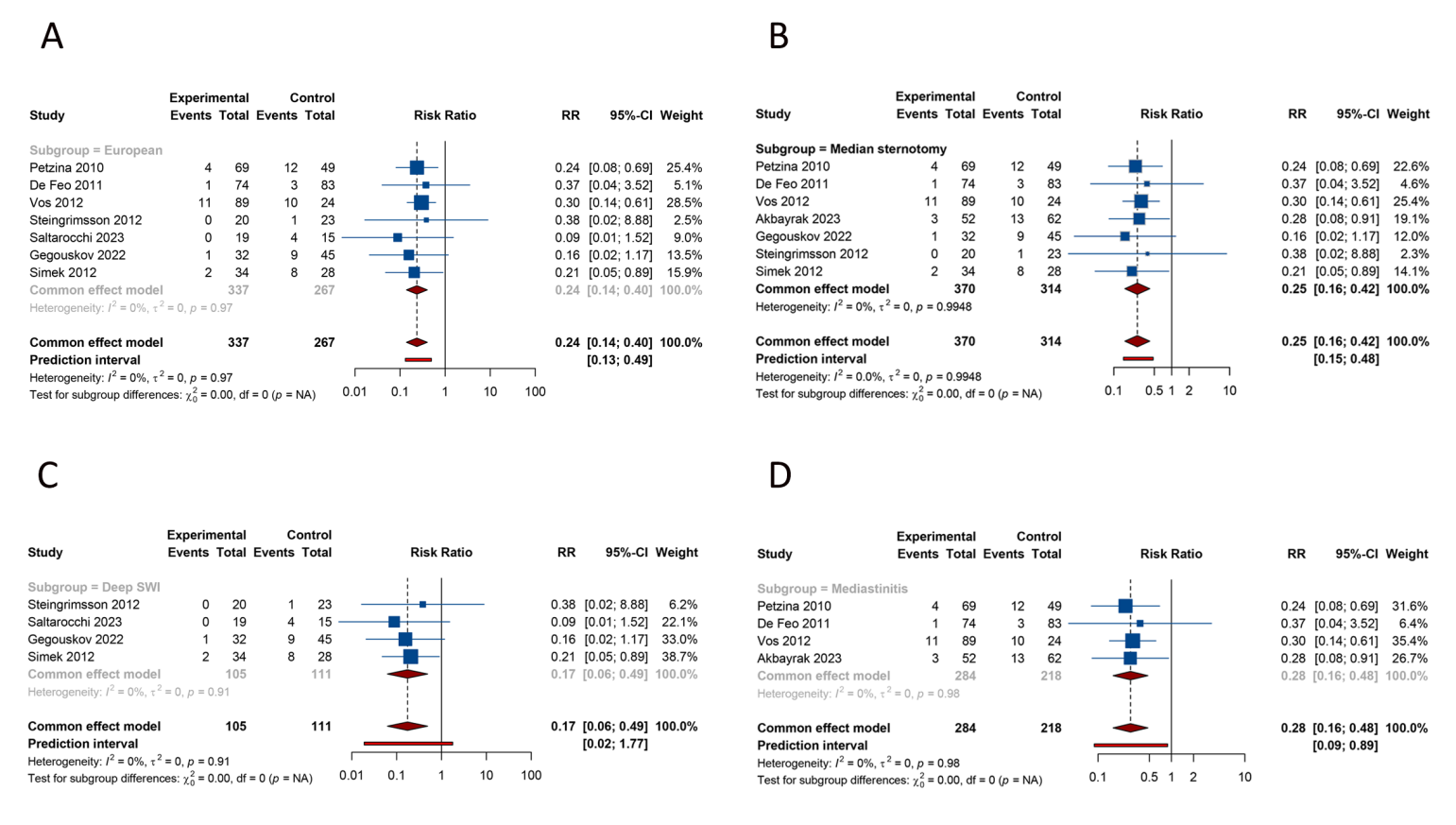


**Fig S3.** Subgroup analysis of ICU stay after negative pressure wound therapy versus conventional wound care for sternal wound infection (SWI). (A) Subgroup = European; (B) Subgroup = Median sternotomy; (C) Subgroup = Deep SWI; (D) Subgroup = Mediastinitis.


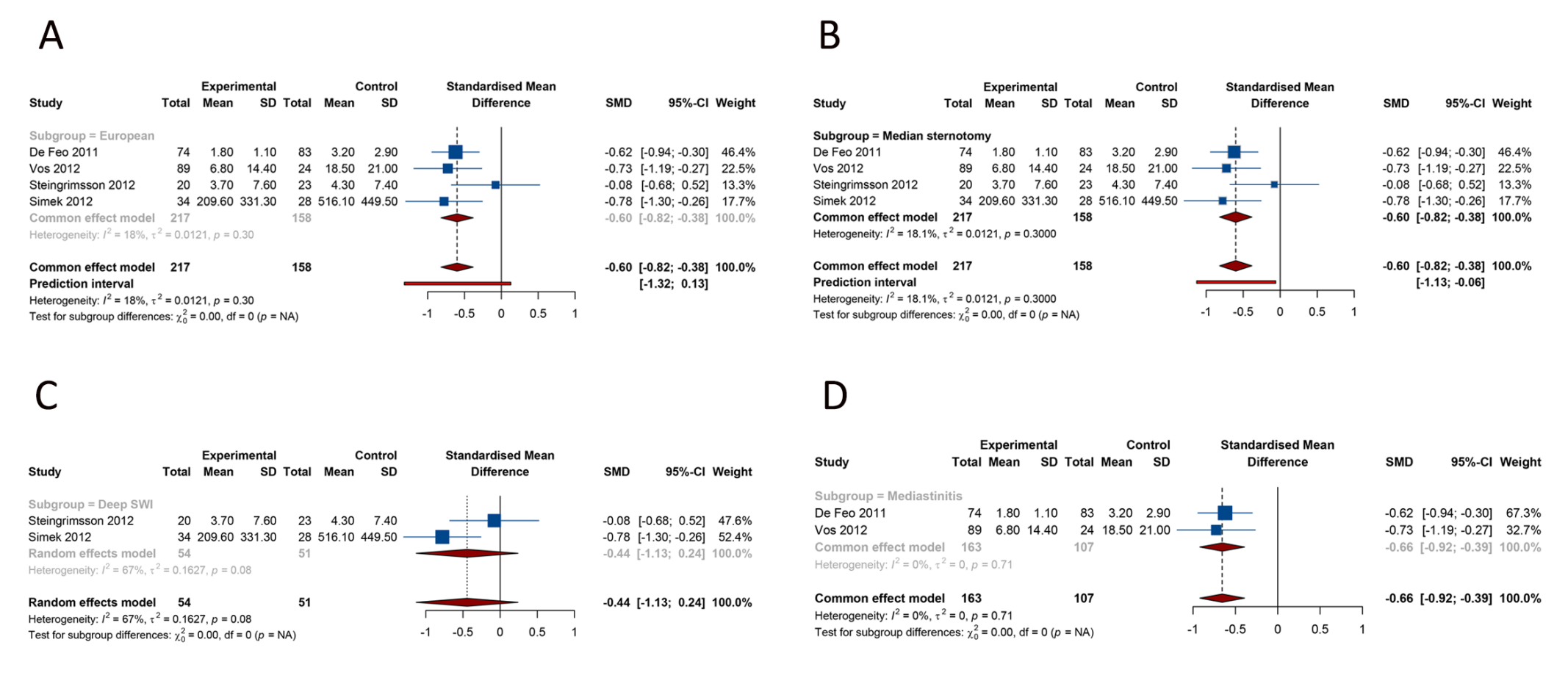


**Fig S4.** Subgroup analysis of hospital stay after negative pressure wound therapy versus conventional wound care for sternal wound infection (SWI). (A) Subgroup = European; (B) Subgroup = Median sternotomy; (C) Subgroup = Deep SWI; (D) Subgroup = Mediastinitis.


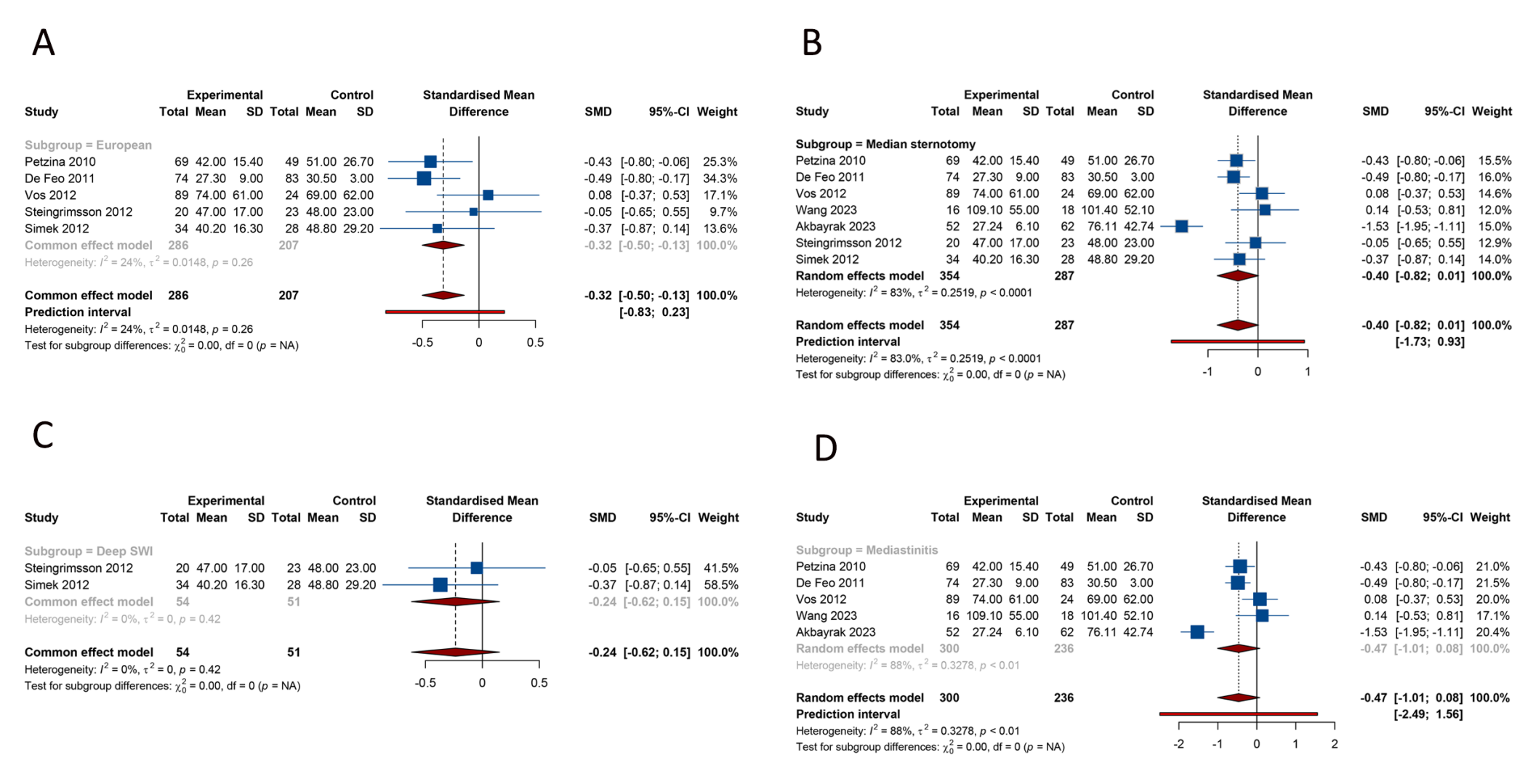


**Fig S5.** Subgroup analysis of treatment duration after negative pressure wound therapy versus conventional wound care for sternal wound infection. (A) Subgroup = European; (B) Subgroup = Median sternotomy; (C) Subgroup = Mediastinitis.


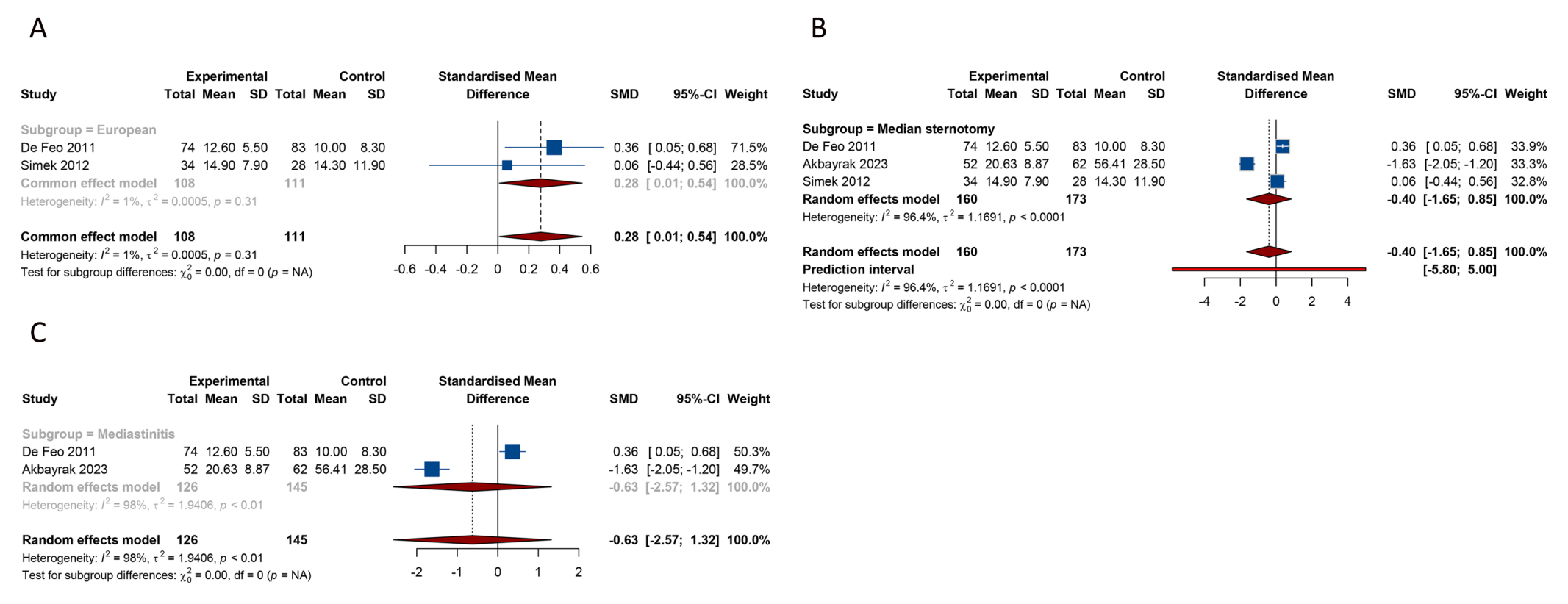


**Fig S6.** Sensitivity analysis on (A) sternal wound reinfection rate, (B) in-hospital mortality, and (C) length of hospital stay after negative pressure wound therapy versus conventional wound care for SWI.


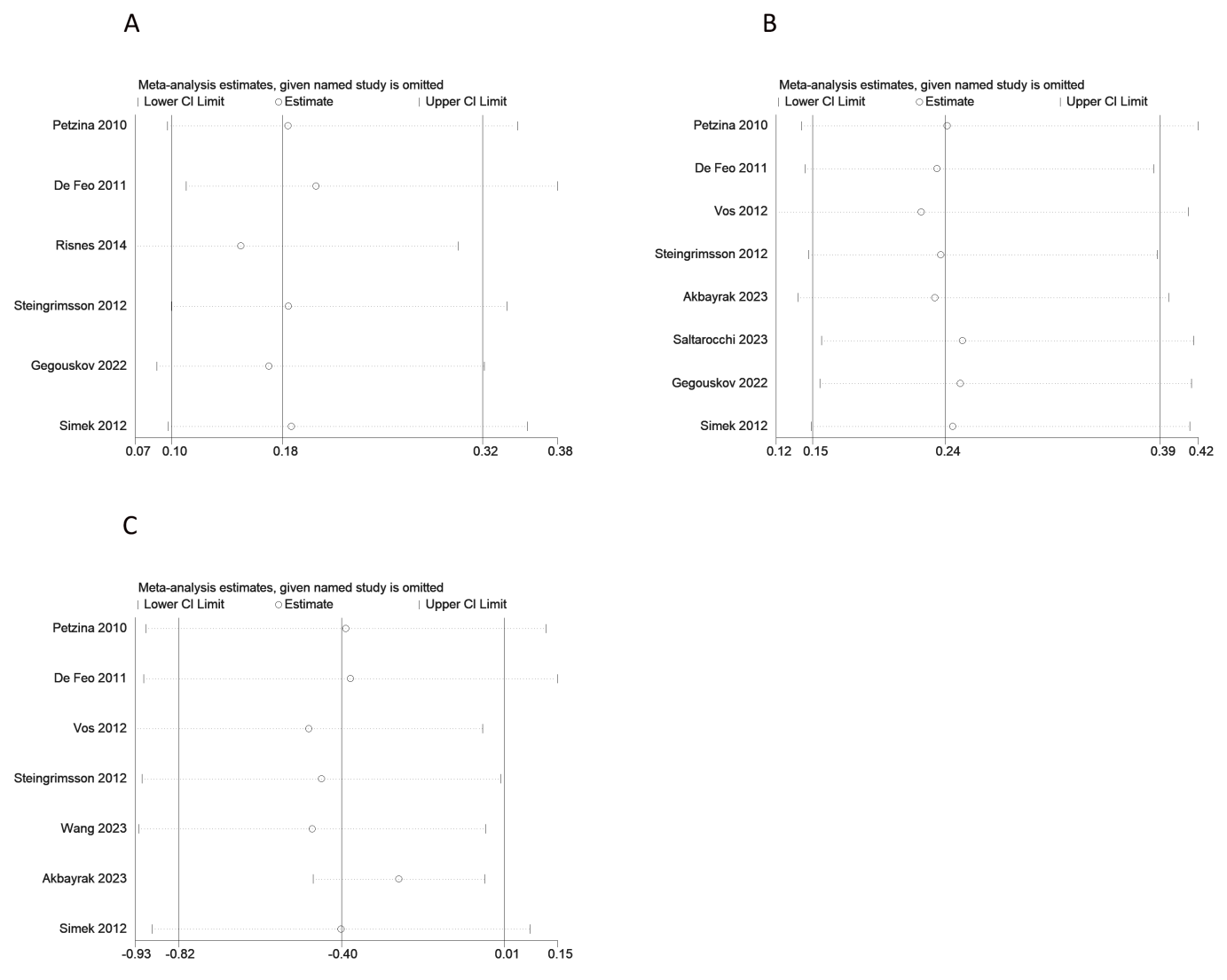


**Fig S7.** Funnel plots of (A) sternal wound reinfection rate, (B) in-hospital mortality, and (C) length of hospital stay after negative pressure wound therapy versus conventional wound care for SWI.


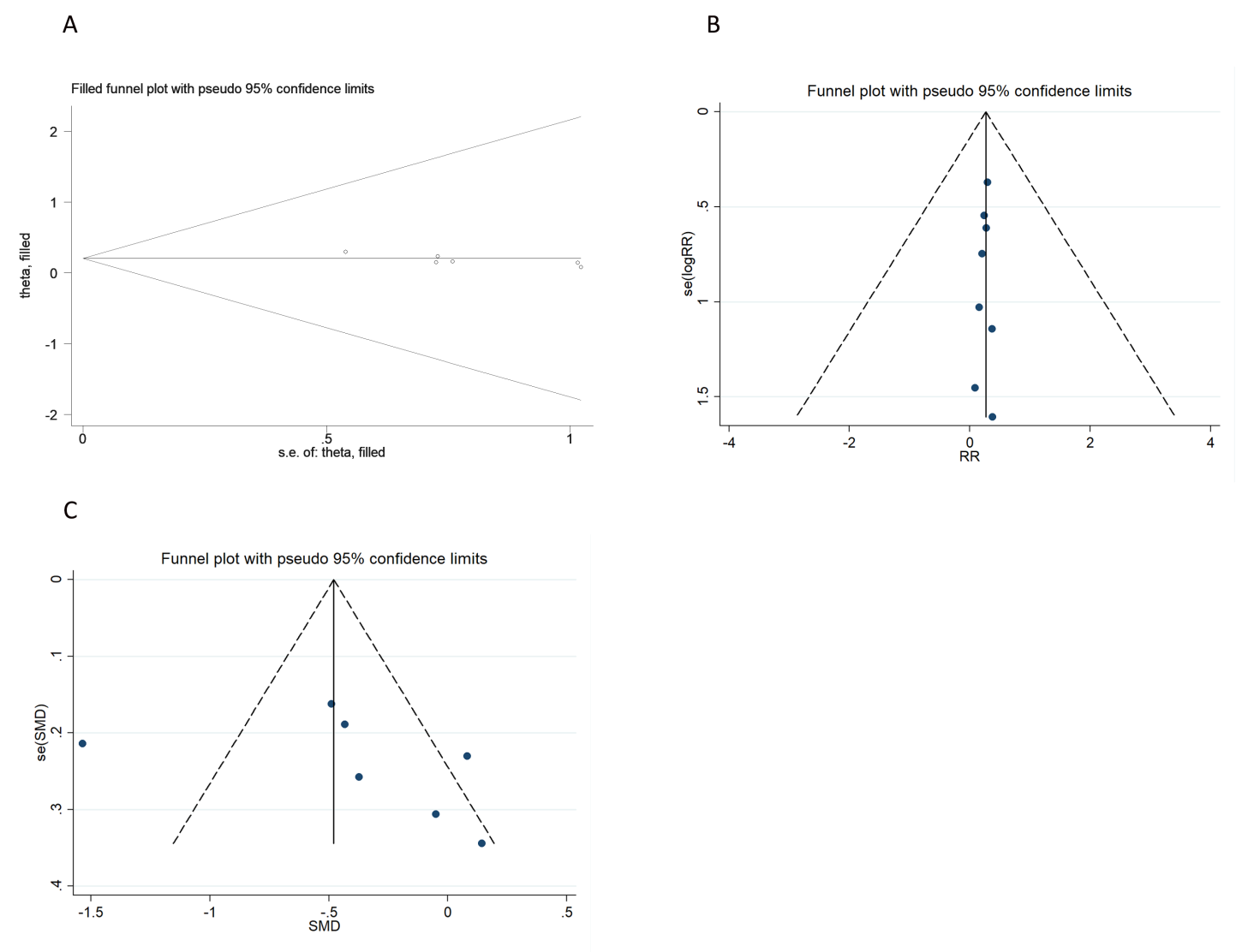

Supplement: S3 File — (DOCX) [file pone.0328771.s004.docx]
